# Assessment of testicular self-examination awareness and practice among adult males in Ajman, United Arab Emirates: A cross-sectional study

**DOI:** 10.1371/journal.pone.0326919

**Published:** 2025-06-25

**Authors:** Abubakker Siddiq Mohamed Hameed, Mohammed Nayeem Shaji, Prasanna Appiya Premvignesh, Janet Chichu James, Miral Salama, Manda Venkatramana, Liju Susan Mathew, Neema Halfan, Anusha Sreejith, Jayadevan Sreedharan, Jayakumary Muttappallymyalil

**Affiliations:** 1 Higher Diploma in Pre-clinical Sciences Program, Gulf Medical University, Ajman, United Arab Emirates; 2 Bachelor of Biomedical Science Program, Gulf Medical University, Ajman, United Arab Emirates; 3 Department of Biomedical Sciences, Gulf Medical University, Ajman, United Arab Emirates; 4 Consultant Surgeon, Thumbay Hospital, Ajman, United Arab Emirates; 5 Department of Community Medicine, Gulf Medical University, Ajman, United Arab Emirates; 6 Thumbay Institute of Population Health, College of Medicine, Gulf Medical University, Ajman, United Arab Emirates; West China Hospital of Sichuan University, CHINA

## Abstract

**Introduction:**

Testicular cancer (TC) is one of the most common cancers affecting men between the ages of 15 and 40 years. Testicular self-examination (TSE) is an effective tool for the early detection of TC, significantly improving survival rates. This study aimed to assess the level of awareness and practice of TSE among adult males in Ajman, United Arab Emirates (UAE).

**Methods:**

A descriptive cross-sectional study was conducted among 1031 adult men residing in Ajman, UAE, using a structured self-administered and interviewer-administered questionnaire. Descriptive statistics were used to summarize the data, and Chi-square tests were applied to assess associations between awareness and practice of TSE with socio-demographic variables.

**Results:**

The mean age of participants was 34.9 ± 9.8 years. Overall, 212 participants (20.6%) reported being aware of TSE. Among those who were aware, 106 individuals (50%) reported practicing TSE.

**Conclusion:**

The findings highlight a low level of awareness and practice of TSE among men in Ajman. There is a critical need for targeted health campaigns and educational interventions to promote knowledge and practice of TSE. Healthcare providers should play a proactive role in raising awareness about TC and the importance of regular self-examination for early detection.

## Introduction

Testicular cancer (TC) accounts for approximately 1% of all male cancers [1], yet it remains the most common malignancy among males aged 15–40 years globally [1]. Despite its relatively low lifetime prevalence of 0.4%, TC carries a significant burden due to its early age of onset and the associated social and psychological impacts on young men [2]. The majority of TC cases are malignant, with seminomas comprising approximately 56% of diagnoses [1]. In the United Kingdom, it is estimated that 1 in 220 men are at risk of developing TC, with the highest incidence observed among men aged 30–34 years between 2017 and 2019 [3]. The risk of developing TC can be attributed to numerous environmental and genetic factors of varying kinds, including a family history of TC [2]. Other risk factors include age, race, body size, diet, and associated congenital anomalies are some of the known risk factors of TC [2].

Worldwide, the incidence of TC has shown a steady increase. In the United States, age-adjusted incidence rates rose by an average of 0.7% annually between 2012 and 2021, with mortality rates increasing by 2.1% annually between 2013 and 2022 [4]. However, public awareness remains low. A study conducted in Saudi Arabia found that only 26.5% of participants had any knowledge about testicular cancer, highlighting the need for targeted education campaigns [5].

Barriers to early detection of TC are multifactorial. Given the sensitivity of the male genital system, abnormalities often trigger significant emotional, behavioral, and social distress, discouraging help-seeking behavior [6]. Feelings of anxiety and shame may prevent men from approaching healthcare providers for screening or evaluation [7]. Furthermore, fears related to the potential removal of a testicle and perceived threats to masculinity negatively impact self-image and willingness to undergo screening [8,9]. Such psychosocial barriers contribute to delayed diagnosis and worsened outcomes.

Testicular self-examination (TSE) represents a simple, non-invasive, and cost-free method for the early detection of TC [10,11]. TSE is generally recommended once a month for males aged 15 years and older [12]. Nevertheless, anticipated regret—a form of emotional distress linked to the fear of discovering abnormalities—has been identified as a significant psychological barrier to the practice of TSE [13,14]. Screening behaviors are also strongly influenced by an individual’s awareness and knowledge of TC and TSE, their attitudes toward preventive health, and the value placed on health education [12].

In this study, awareness was conceptualized as a participant’s ability to correctly identify testicular self-examination (TSE) as a preventive tool, understand its purpose, and demonstrate basic knowledge of when and how it should be performed. Participants received an awareness score based on their responses to knowledge-based questions about TSE. The median value of the awareness scores was used as the cutoff point to classify participants into groups with either low or good awareness. This data-driven approach allowed for a more accurate and context-specific categorization of awareness levels among the study population.

Early detection of TC through TSE is vital to improve survival rates [11]. However, awareness and practice of preventive health behaviors remain limited among men in the United Arab Emirates (UAE), partly due to cultural stigmas surrounding male reproductive health [15]. Ajman, although rapidly developing in terms of healthcare infrastructure, remains underrepresented in region-specific public health research, especially concerning male-specific cancers [16]. Previous studies have indicated that cancer awareness initiatives in the UAE have predominantly focused on larger emirates such as Abu Dhabi and Dubai, with comparatively fewer targeted interventions in smaller emirates like Ajman [17]. Additionally, a study on healthcare behavior in the UAE highlighted that men are less likely than women to engage in preventive screening practices, further emphasizing the need for localized studies to understand barriers to early cancer detection [18].

This study adds to the existing literature by addressing the specific gap in region-based knowledge regarding TSE awareness in Ajman, a smaller emirate of the UAE. Despite the rapid development in healthcare, there remains limited data on the unique sociocultural factors influencing TSE knowledge and practices in Ajman, which may differ from other areas of the UAE. By identifying local barriers and knowledge gaps, our findings offer valuable insights for policymakers to design culturally relevant and region-specific health education programs. These findings can inform the development of targeted awareness campaigns and contribute to improving early detection practices in this underserved area.

Given these factors, this study aims to assess the level of awareness, knowledge, and practice of TSE among adult males in Ajman, United Arab Emirates. By identifying prevailing knowledge gaps, behavioral patterns, and sociocultural barriers, the findings are intended to inform targeted public health strategies. These results may contribute to the development of community-based education programs and health promotion initiatives within the local context [19].

## Methods

### Study design, setting, and sampling

This study employed a cross-sectional design and was conducted in Ajman, United Arab Emirates, between November 2023 and March 2024. Adult males aged above 20 years were included. Participants aged 15–17 required parental consent, and among the participants recruited, none of them were below the age of 20 years. This clarification is provided to address ethical considerations and to contextualize the final study population, given the higher prevalence of testicular cancer in the 15–40-year age group. A total of 1031 participants were involved in the study. The sample size was determined using convenience sampling.

### Data collection procedure

A self-administered questionnaire, adapted from a previously validated study conducted among male undergraduates in Nigeria, was used as the data collection instrument [20]. The questionnaire consisted of 38 items organized into five sections: Section A captured socio-demographic data, while Sections B through E assessed awareness of testicular cancer (TC) and TSE, practice of TSE, knowledge of the steps involved in TSE, and factors influencing its practice.

Participants were recruited from various community-based locations in Ajman, including a university, hospital, and public areas. Eligible male participants were approached by the researchers, who explained the objectives of the study and obtained informed consent prior to participation. A pilot test was conducted among 5 participants to ensure clarity and comprehension of the questionnaire prior to the main data collection. Necessary modifications were made based on pilot feedback.

The questionnaire was designed for self-administration; however, interviewer assistance was provided when needed to ensure participants fully understood the questions and to minimize missing or incomplete responses. Given the sensitive nature of the topic and the setting of data collection, some participants preferred or required help during completion. This approach was intended to enhance data quality and participant engagement. While interviewer assistance could potentially introduce some social desirability bias, it likely contributed to a higher response rate and more accurate data collection.

Although convenience sampling was used, the response rate of 94.7% was calculated based on the number of individuals approached (n = 1088) who agreed to participate (n = 1031) during data collection.

The questionnaire was administered in an interviewer-assisted format: researchers asked each question verbally and recorded participants’ responses directly into a digital form using Google Forms on mobile devices. To facilitate better comprehension, the questionnaire was provided in the participants’ preferred native languages (Arabic, Hindi, Urdu, Malayalam, Tamil, etc.) when necessary. This approach enhanced understanding and improved response accuracy. Anonymity and confidentiality were maintained throughout the study, with no identifying information collected from respondents.

The complete questionnaire used for data collection is provided as Supplementary Material 1

(S1 File) to enhance transparency and replicability.

### Data analysis

Data analysis was conducted using IBM® Statistical Package for Social Sciences (SPSS) (version 29.0). Descriptive statistics were employed to summarize categorical data, while chi-square tests were performed to assess the association between the level of awareness and the practice of TSE.

Age was categorized using a 40-year cutoff to reflect a conventional public health threshold distinguishing younger from middle-aged adults. This stratification allowed us to explore potential behavioral differences in testicular self-examination awareness across life stages.

A p-value less than 0.05 was considered statistically significant, in accordance with conventional standards for hypothesis testing in biomedical research.

### Ethics statement

The study was approved by the Institutional Review Board (IRB) of Gulf Medical University (Approval Ref. no. IRB/COM/FAC/29/June-2022) in accordance with the Declaration of Helsinki. Informed consent was obtained electronically through the first question of the digital form. Participants were assured of confidentiality of their responses and that data would be used solely for research purposes. No compensation was provided. The research team adhered to all ethical standards and institutional guidelines.

## Results

A total of 1031 responses were analyzed. The median age of participants was 30 ± 15 years. The majority were from the Southeast Asia Region (54%), and more than two-thirds were married (75.5%). Over half of the individuals were graduates (50.9%), and most participants were employed (81.9%). The socio-demographic characteristics of participants are presented in Table 1.

**Table 1:** Baseline characteristics of participants, n = 1031.

Continuous Variables	Median	SD or IQR
Age(Years)	30	15
Categorical Variables	Group	No(%)
Age group	Less than 40 Years	752 (72.9%)
	Greater than & equal to 40 years	279 (27.1%)
Nationality	South-East Asia Region	557 (54%)
	Eastern Mediterranean Region	415 (40.3%)
	Others	59 (5.3%)
Marital status	Single	253 (24.5%)
	Married	778 (75.5%)
Education	Highschool	330 (32.2%)
	Graduate	525 (50.9%)
	Post-graduate and above	176 (17.1%)
Employment Status	Yes	844 (81.9%)
	No	187 (18.1%)

### Awareness of TSE

Participants received one point for each correct answer and zero points for incorrect answers on the awareness questions. An awareness score was calculated for each participant. Participants scoring more than half of the total points were categorized as having a good level of awareness, while those scoring less were categorized as having a poor level of awareness.

**Fig 1:**
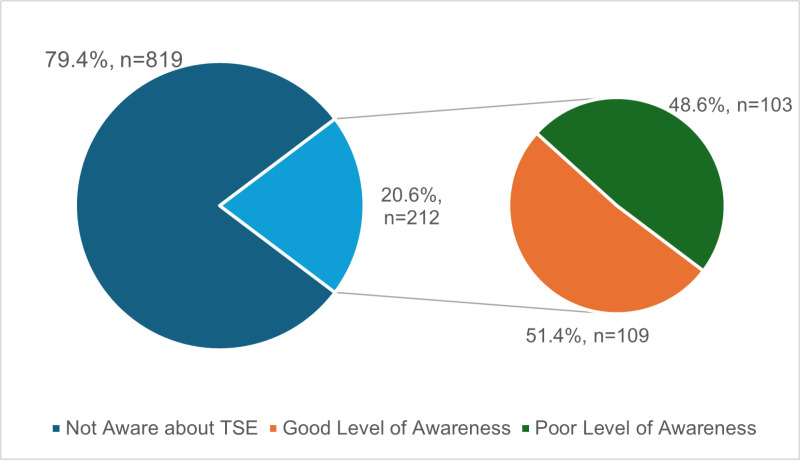
Awareness among the participants, n = 1031.

As presented in Fig 1 – Of the 1031 participants, 212 (20.6%) reported awareness of TSE, while the remaining 819 (79.4%) had never heard of it. Among those aware, 109 (51.4%) demonstrated a good level of awareness based on their correct responses to TSE-related questions, while 103 (48.6%) exhibited poor awareness.

Among them, 90.6% (n = 192) believed that TSE could help in the early diagnosis of TC. When asked about the appropriate age to initiate TSE, 49.1% (n = 104) indicated between 16–20 years, 11.5% (n = 24) mentioned 21–25 years, 5.7% (n = 12) indicated 26–30 years, and 15.6% (n = 33) mentioned 31 years and above. The distribution of participants according to their level of awareness is shown in Fig 1. A majority demonstrated a poor level of awareness.

### Practice of TSE

Most of the participants, 925; have never performed TSE. Among the 212 participants who heard about TSE, only 10.3% (n = 102) reporting practicing it and of those, only 37.3% (n = 79) practiced it regularly. A little higher than one-third 34.4% (n = 73) knew the steps involved in TSE. 10.8% (n = 23) practiced weekly, 25% (n = 53) practiced monthly, 15.1% (n = 32) Yearly. One-sixth 15.6% (n = 33) of those who performed felt that TSE is time-consuming. Even a lesser percentage 12.3% (n = 26) complained of being difficult to perform.

### Knowledge of TSE steps

Participants who had heard about TSE (n = 212) were asked specific questions regarding the correct steps involved. Table 2 summarizes their responses.

**Table 2:** Awareness of steps in performing TSE, n = 212.

Statements	Response	No.
*Correct statements*		
Stand in front of the mirror and look for swelling on the scrotum	Yes	92 (43.4%)
Using both hands, the scrotum should be gently lifted so that the area underneath can be checked	Yes	102 (48.1%)
The index and the middle finger should be placed under each testicle with the thumb on top	Yes	93 (43.9%)
The testes should be examined one at a time	Yes	106 (50%)
Roll each testicle between fingers and thumb	Yes	90 (42.5%)
Feel for lumps of any size	Yes	106 (50%)
*Incorrect statements*		
Lie on the bed and look for swelling on the scrotum	No	66 (31.1%)
Use both hands to examine both testes together as one	No	49 (23.1%)
Roll each testis with the thumb alone	No	56 (26.4%)

### Factors affecting the practice of TSE

**Fig 2:**
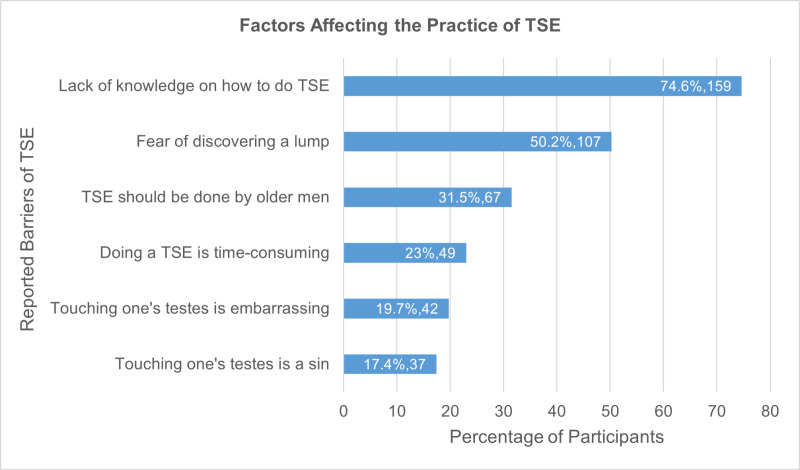
Factors affecting the practice of TSE, n = 212. The horizontal bar graph depicts the frequency and percentage of different perceived barriers to TSE practice.

Among the 212 participants aware of TSE, the most commonly reported barrier was a lack of knowledge on how to perform it, followed by fear of discovering a lump and misconceptions regarding the appropriate age to perform TSE. Other barriers, such as embarrassment, time constraints, and religious concerns, were less frequently reported (Fig 2).

### Awareness level and practice: Analytical findings

A Chi-square test was conducted to assess the association between the level of awareness and the practice of TSE. The results in Table 3 indicate a significant association (p < 0.001), with individuals having a good level of awareness being more likely to practice TSE.

**Table 3:** Awareness of steps in performing TSE, n = 212.

Variable	Group	Practice		p-value
		No	Yes	
**Level of Awareness**	Poor level of awareness	74 (71.8%)	29 (28.2%)	<0.001
	Good level of awareness	32 (29.4%)	77 (70.6%)	

### Socio-demographic factors and awareness levels: Analysis results

Demographic factors showed significant associations with the level of TSE awareness (Table 4). Nationality, education level, relationship status, and employment status were all significantly linked to awareness (p < 0.01). Participants from the Eastern Mediterranean Region, those with high school education, single individuals, and unemployed participants demonstrated higher levels of awareness. No significant association was observed with age (p = 0.081).

**Table 4:** Distribution of socio-demographic characteristics by level of awareness regarding testicular self-examination (n = 212).

Variable	Group	Level of Awareness		p-value
		Poor Level of Awareness	Good Level of Awareness	
**Age**	Less than 40 years	77 (45.6%)	92 (54.4%)	0.081
	Greater than & equal to 40 years	26 (60.5%)	17 (39.5%)	
**Nationality**	South-East Asia Region	59 (59.6%)	40 (40.4%)	0.004
	Eastern Mediterranean Region	39 (41.9%)	54 (58.1%)	
	Others	5 (25%)	15 (75%)	
**Education**	Highschool	28 (35.9%)	50 (64.1%)	0.004
	Graduate	48 (62.3%)	29 (37.7%)	
	Post-Graduate and above	27 (47.4%)	30 (52.6%)	
**Relationship**	Single	35 (36.5%)	61 (63.5%)	0.001
	Married	68 (58.6%)	48 (41.4%)	
**Employment Status**	Employed	77 (59.2%)	53 (40.8%)	<0.001
	Unemployed	26 (31.7%)	56 (68.3%)	

### Perceived barriers and awareness: Analysis results

Several perceived barriers were significantly associated with awareness levels (Table 5). Participants who perceived TSE as embarrassing, sinful, or time-consuming, or who feared discovering a lump, were less likely to have good awareness (p < 0.01). However, lack of knowledge on how to perform TSE was not significantly associated with awareness (p = 0.383).

**Table 5:** Distribution of perceived barriers according to awareness level of testicular self-examination (n = 212).

Variable	Group	Level of Awareness		p-value
		Poor Level of Awareness	Good Level of Awareness	
**Touching one’s own testes is embarrassing?**	No	93 (54.7%)	77 (45.3%)	<0.001
	Yes	10 (23.8%)	32 (76.2%)	
**Fear of discovering a lump?**	No	63 (60%)	42 (40%)	0.001
	Yes	40 (37.4%)	67 (62.6%)	
**Lack of knowledge on how to do Testicular Self–Examination?**	No	23 (43.4%)	30 (56.6%)	0.383
	Yes	80 (50.3%)	79 (49.7%)	
**Touching one’s testes is a sin?**	No	97 (55.4%)	78 (44.6%)	<0.001
	Yes	6 (16.2%)	31 (83.8%)	
**Doing Testicular Self–Examination is time-consuming?**	No	93 (57.1%)	70 (42.9%)	<0.001
	Yes	10 (20.4%)	39 (79.6%)	
**Testicular Self–Examination should be done by older men?**	No	89 (61.4%)	56 (38.6%)	<0.001
	Yes	14 (20.9%)	53 (79.1%)	

### Socio-demographic factors and practice: Analysis results

Demographic factors did not show significant associations with the practice of TSE (Table 6). Age, nationality, education level, relationship status, and employment status were not significantly linked to practice behavior (p > 0.05). While participants aged ≥ 40 years showed a slightly higher proportion of TSE practice (53.5%) compared to those under 40 (49.1%), the difference was not statistically significant (p = 0.608). A relatively higher practice rate was also observed among participants from the Eastern Mediterranean Region (59.1%) compared to other regions; however, this difference was not statistically significant (p = 0.061).

**Table 6:** Distribution of socio-demographic characteristics by testicular self-examination practice (n = 212).

Variable	Group	Practice		p-value
		No	Yes	
**Age**	Less than 40 years	86 (50.9%)	83 (49.1%)	0.608
	Greater than or equal to 40 years	20 (46.5%)	23 (53.5%)	
**Nationality**	South-East Asia Region	57 (57.6%)	42 (42.4%)	0.061
	Eastern Mediterranean Region	38 (40.9%)	55 (59.1%)	
	Others	11 (55%)	9 (45%)	
**Education**	Highschool	36 (46.2%)	42 (53.8%)	0.291
	Graduate	44 (57.1%)	33 (42.9%)	
	Post-Graduate and above	26 (45.6%)	31 (54.4%)	
**Relationship Status**	Single	48 (50%)	48 (50%)	1
	Married	58 (50%)	58 (50%)	
**Employment Status**	Employed	65 (50%)	65 (50%)	1
	Unemployed	41 (50%)	41 (50%)	

There were no notable differences in TSE practice observed across education levels, marital status, or employment status. Since chi-square tests did not reveal any significant associations with TSE practice, further statistical analyses, such as logistic regression, were not conducted.

### Perceived barriers and practice: Analysis results

Perceived barriers were not significantly associated with the practice of TSE (Table 7). Factors such as embarrassment, fear of discovering a lump, lack of knowledge, the belief that touching one’s testes is sinful, and the perception that TSE is time-consuming showed no significant associations with practice behavior (p > 0.05 for all). Although participants who believed that TSE should be performed only by older men demonstrated a higher practice rate (59.7%), this association was borderline and did not reach statistical significance (p = 0.055).

**Table 7:** Distribution of perceived barriers by practice of testicular self-examination (n = 212).

Variable	Group	Practice		p-value
		No	Yes	
**Touching one’s own testes is embarrassing?**	No	85 (50%)	85 (50%)	1
	Yes	21 (50%)	21 (50%)	
**Fear of discovering a lump?**	No	54 (51.4%)	51 (48.6%)	0.68
	Yes	52 (48.6%)	55 (51.4%)	
**Lack of knowledge on how to do TSE?**	No	26 (49.1%)	27 (50.9%)	0.874
	Yes	80 (50.3%)	79 (49.7%)	
**Touching one’s testes is a sin?**	No	90 (51.4%)	85 (48.6%)	0.366
	Yes	16 (43.2%)	21 (56.8%)	
**Doing TSE is time-consuming?**	No	84 (51.5%)	79 (48.5%)	0.415
	Yes	22 (44.9%)	27 (55.1%)	
**TSE should be done by older men?**	No	79 (54.5%)	66 (45.5%)	0.055
	Yes	27 (40.3%)	40 (59.7%)	

### Logistic regression analysis of awareness levels by sociodemographic variables

Sociodemographic factors such as education, nationality, relationship status, and employment were assessed (Table 8). After adjusting for covariates, education and nationality showed significant associations with good awareness. Compared to participants with post-graduate education, those who were graduates had significantly lower odds of demonstrating good awareness (adjusted OR 0.41, 95% CI 0.19–0.87, P = 0.021), while participants with high school education did not show a statistically significant association. With regard to Nationality; participants from the EMR region (adjusted OR 2.27, 95% CI 1.23–4.19, P = 0.009) and those categorized as Others (adjusted OR 4.16, 95% CI 1.31–13.20, P = 0.016) were significantly more likely to have good awareness compared to those from SEAR. Relationship status did not show a significant association with awareness after adjustment (P = 0.591). Although employed participants had higher odds of good awareness (adjusted OR 2.47, 95% CI 0.98–6.26), this association did not reach statistical significance (P = 0.056). These findings highlight nationality and educational attainment as independent predictors of good awareness within the studied population.

**Table 8:** Predictors of awareness based on sociodemographic characteristics (n = 212).

Variable	Group	Crude OR (CI 95%)	Adjusted OR (CI 95%)	P-value (Adjusted)
Education	Highschool	1.607 (0.801–3.223)	0.686 (0.261–1.803)	0.445
	Graduate	0.544 (0.271–1.089)	0.411 (0.194–0.874)	0.021
	Post-Graduate and above	1	1	–
Nationality	South-East Asia Region	1	1	–
	Eastern Mediterranean Region	2.042 (1.149–3.630)	2.268 (1.228–4.188)	0.009
	Others	4.425 (1.490–13.146)	4.16 (1.311–13.203)	0.016
Relationship	Single	2.469 (1.416–4.306)	1.267 (0.535–2.999)	0.591
	Married	1	1	–
Employment Status	Employed	3.129 (1.749–5.600)	2.474 (0.978–6.261)	0.056
	Unemployed	1	1	–

## Discussion

This study assessed the awareness and practice of TSE among 1031 adult males. The findings revealed a markedly low level of both awareness and practice. Only 20.6% of participants had ever heard of TSE, and among them, less than half (49.1%) knew the recommended age to begin performing TSE. Awareness of TSE was generally low among the participants. Even among those who had heard of TSE, only 10.3% reported ever performing it, and just 37.3% of those did so regularly, highlighting a significant gap between awareness and actual practice.

A significant association was observed between the level of awareness and the likelihood of practicing TSE. Good awareness significantly increased the likelihood of practice. Certain socio-demographic factors, including nationality, education, marital status, and employment status, were significantly associated with awareness levels, but none of these factors were significantly linked to TSE practice. Common perceived barriers to TSE included lack of knowledge, fear of discovering a lump, and misconceptions regarding age, while embarrassment and religious concerns were less frequently reported. Interestingly, many of these barriers, such as perceiving TSE as sinful, embarrassing, or time-consuming, were significantly associated with lower awareness, but not with practice behavior.

Multivariable logistic regression analysis revealed that participants’ educational attainment and nationality were significant predictors of good awareness of TSE. Graduates had significantly lower odds of good awareness compared to post-graduates, while those from the EMR and Other regions were more likely to be aware than participants from SEAR. Relationship status and employment did not show statistically significant associations after adjusting for confounders.

A key strength of our study lies in its focus on a general population sample in the Ajman, UAE, a region where research on TSE remains scarce. This contrasts with existing studies that primarily target student or academic populations, allowing our findings to offer broader insight into public awareness and behavior surrounding TSE. The structured questionnaire, tailored specifically to assess basic awareness and self-reported practice rather than psychosocial or behavioral constructs, was appropriately chosen given the exploratory nature of our research and the cultural sensitivity of the topic.

Our study revealed that 79.4% of participants had never heard of TSE, a proportion significantly higher than that reported in Nigeria (46%) and Ethiopia (32%) [20,21]. This underscores a pronounced gap in awareness within our population and highlights the urgent need for targeted public health interventions. A study among young males in Poland reported that while 70–100% had heard about TC, only 30–50% recognized its risk factors properly [22]. Although our study did not specifically assess knowledge of TC risk factors, the low awareness and practice of TSE observed among our participants reflect a similarly concerning lack of attention to testicular health. In this context, while validated scales such as the SEPTSES (Rew et al., 2005) exist for assessing self-efficacy and psychosocial constructs related to TSE, our study specifically aimed to evaluate basic awareness and self-reported practice behaviors among the general population. A study among university students in Turkey revealed that a large majority were not knowledgeable about TC and did not know how to perform TSE, with 72.4% of male subjects having not heard about TSE previously and 89.4% not knowing how to do it [23]. Our findings are consistent with those of the Turkish study among university students, further demonstrating that even within academic environments, there is a substantial lack of practical knowledge about TSE. This suggests that university settings may offer valuable opportunities for promoting awareness of TSE and testicular health among youth; however, it is essential that awareness efforts also reach non-university populations to ensure equitable access to information. In addition, among the 212 individuals aware of TSE, only 53 (24.4%) were aware of the process and steps involved, indicating the need for education initiatives to improve understanding.

These comparisons highlight a consistent trend across multiple regions, awareness does not necessarily translate into knowledge or effective practice. Of the 212 participants aware of TSE in our study, only half (50%) reported practicing it. However, reported frequency and accuracy varied, suggesting that self-reported practice may overestimate effective behavior. This is consistent with findings from Nigeria, where only 11.6% of respondents practiced TSE regularly [20], and Uganda, where just 23.6% of students engaged in TSE [24]. A systematic review of 25 studies also emphasized that TSE knowledge and practice remain low in many developing countries, often due to poor public awareness and education [25].

A significant strength of our study is its exploration of perceived barriers to TSE such as fear and embarrassment mirrored those in the systematic review, pointing to widespread psychological and cultural hindrances that need to be addressed through tailored educational efforts. Likewise, in Turkey, 89.4% of university students did not know how to perform TSE, and 90.6% had not received any form of training [26]. The Polish study also highlighted low awareness and infrequent practice of TSE among young males [21]. Another study in academic settings showed that sociocultural norms, religious beliefs, and fear of detecting lumps were key barriers to TSE practice, although many respondents expressed willingness to adopt TSE following proper guidance [27]. These findings reinforce the need for culturally sensitive and skill-based educational interventions to enhance both the knowledge and practice of TSE. Moreover, factors such as time constraints, embarrassment, and fear of discovering abnormality with TSE were identified as barriers to regular practice. For instance, 23% of our participants who heard about TSE reported TSE as time-consuming, while 19.7% felt embarrassed about it. These findings are in line with those from Nigeria, Uganda, and Poland, where similar psychological and cultural hindrances were identified as major barriers to practice [20,22,24]. The Turkish university study and systematic review also echoed these themes, highlighting fear, neglect, and misconceptions, such as the belief that TSE is only for older men as pervasive deterrents [23,25].

A further strength is our identification of sociocultural misconceptions: 31.5% of participants believed TSE is only necessary for older individuals. Such beliefs were similarly reported in Poland and Turkey, where cultural norms, religious beliefs, and stigma were common reasons for avoidance [22,23], A Nigerian undergraduate study highlighted the need for health promotion and interventions to address poor attitudes and misconceptions about TSE [27]. Although most respondents did not find TSE sinful or shameful, fear and lack of knowledge emerged as dominant themes, suggesting that awareness campaigns must also address cultural and emotional barriers.

Nonetheless, a notable limitation is the reliance on self-reported data, which may be subject to recall and social desirability biases. Additionally, our study did not assess participants’ actual competence or accuracy in performing TSE, an important factor in early cancer detection. While other studies have used validated tools like the SEPTSES (Rew et al., 2005). An Iranian study showed that educational interventions based on health belief models and social support significantly improved knowledge and TSE practices [28]. The Health Belief Scale study suggested that using validated tools to assess health beliefs can help tailor interventions to improve TSE practice by addressing perceived barriers and enhancing self-efficacy [29]. Another Turkish study recommended improvements in medical education to ensure that future healthcare professionals are adequately trained in TSE and TC detection techniques [30]. Our focus was on establishing a foundational understanding of awareness and behavior, which may limit deeper behavioral insights.

Another limitation is the lack of subgroup analysis, particularly among healthcare students or professionals, who could serve as key advocates for awareness. Studies in Poland and Nigeria noted that educational level and field of study significantly influenced TSE awareness and practice [22,27]. Despite these limitations, our findings align with literature emphasizing the need for culturally sensitive, skill-based, and age-appropriate interventions. As suggested by the Polish and Ugandan studies, mass media and educational curricula can be leveraged to promote accurate information about TC and TSE [22,24]. Participants in other studies have expressed willingness to learn from healthcare professionals [23], and evidence from Iran supports the effectiveness of health belief model-based interventions in improving TSE practice [28]. These insights reinforce the broader applicability and significance of our study’s conclusions for public health planning in the region.

This study underscores the pressing need for improved testicular health literacy in Ajman, revealing alarmingly low levels of awareness and practice of TSE, even among relatively young and educated individuals. The findings suggest that despite the UAE’s overall healthcare advancements, specific communities like Ajman remain underserved in terms of preventive health education, particularly concerning male reproductive health. The results hold significant implications for clinicians and policymakers: there is a clear need to implement culturally sensitive, age-appropriate, and skill-based educational interventions that address prevalent misconceptions, sociocultural barriers, and psychological deterrents such as fear and embarrassment. Healthcare providers should be trained to initiate conversations about TSE during routine consultations, and public health campaigns—leveraging schools, universities, and social media—should be prioritized to normalize the practice and promote early detection of testicular cancer. Furthermore, integrating TSE education into health curricula and community outreach programs could empower young men to take charge of their reproductive health.

Despite its contributions, the study raises important questions that warrant further investigation. For instance, the relationship between awareness and actual TSE proficiency remains unclear, as does the long-term impact of educational interventions in altering behavior. Future research should aim to evaluate the effectiveness of targeted interventions, explore differences across various regions and demographic groups in the UAE, and consider the role of religious and cultural beliefs more deeply. Using validated tools to assess health beliefs and self-efficacy may also enhance future studies. By addressing these unanswered questions, future work can guide the design of more impactful and sustainable strategies for improving testicular cancer prevention efforts in the region.

## Conclusion

The study highlights a concerning lack of awareness and low practice of TSE among men in Ajman, United Arab Emirates. Factors such as lack of awareness, fear of discovering a lump, and the belief that only older men should perform TSE contribute to this gap. Action is needed to address these challenges.

Comprehensive health campaigns and targeted awareness initiatives are crucial to disseminating accurate information about TSE and its importance in TC prevention. In addition, integrating TSE education into school curricula and workplace health programs can facilitate long-term behavioural changes.

By fostering open dialogue and culturally sensitive approaches, we can empower men to prioritize their health and promote early detection of TC. Targeted interventions addressing these barriers may enhance TSE practice in the region, ultimately contributing to earlier detection of testicular cancer and improved health outcomes among men in Ajman.

## Supporting information

S1 File Questionnaire. (DOCX)

S2 File Data. (XLSX)
