# Supplementary material for: Assessment of testicular self-examination awareness and practice among adult males in Ajman, United Arab Emirates: A cross-sectional study
Source: PLoS One. 2025 Jun 25;20(6):e0326919. doi: 10.1371/journal.pone.0326919 (PMC12193016; doi:10.1371/journal.pone.0326919)
Supplement: S1 File — (DOCX) [file pone.0326919.s001.docx]

**Questionnaire**

**Level of awareness and practice of testis self-examination among adult males in Ajman United Arab Emirates**

**=================================================================**

1. **Socio-demographic information**
2. Age: …….2. Nationality:………………….3. Education:…………..
3. Occupation:…………….5. Relationship status: Single/Married
4. **Awareness of Testicular Self-Examination**
5. Have you ever heard of testicular self-examination? Yes/No
6. Who should know about testicular self-examination? All men/Male students/Health workers/Everybody/Others:-Please specify………………..
7. Does testicular-self-examination help in the early diagnosis of testicular cancer?

Yes/No

1. Is awareness of testicular self-examination important to men? Yes/No
2. At what age should one start testicular self-examination?....................
3. **Practice of testicular Self-Examination**
4. Do you practice Testicular Self-Examination? Yes/No
5. Do you know the process and steps involved in Testicular Self-Examination? Yes/No
6. When do you practice Testicular Self-Examination? Every week/Every month/Once in a year/I don’t know/ Never performed it
7. When is the best time to perform testicular self-examination? Morning / Afternoon / Evening / Night / Never performed it
8. How would you rate your performance? Excellent/Very good/Good/Fair/Poor/ Never performed it
9. Is Testicular Self–Examination time-consuming? Yes/No/NA
10. Do you practice Testicular Self-Examination regularly? Yes/No/NA
11. Do you feel satisfied with the way you perform Testicular Self-Examination? Yes/No/NA
12. Is Testicular Self-Examination difficult to perform? Yes/No/NA
13. Should Testicular Self-Examination be practiced by only people at risk of testicular cancer? Yes/No/NA
14. How often do you think Testicular Self–Examination should be performed?

Daily/Weekly/Monthly/Annually/When a lump is suspected/I don’t know

1. When last did you perform Testicular Self–Examination? Weeks ago/Months ago/One month/A year ago/Never/1 week /few days ago
2. **Steps for performing** **Testicular Self-Examination**
3. Stand in front of the mirror and look for swelling on the scrotum Yes/No/NA
4. Lie on the bed and look for swelling on the scrotum Yes/No/NA
5. Using both hands, the scrotum should be gently lifted so that the area underneath can be checked Yes/No/NA
6. The index and the middle finger should be placed under each testicle with the thumb on top. Yes/No/NA
7. The testes should be examined one at a time Yes/No/NA
8. Use both hands to examine both testes together as one Yes/No/NA
9. Roll each testis with thumb alone Yes/No/NA
10. Roll each testicle between fingers and thumb Yes/No/NA
11. Feel for lumps of any size Yes/No/NA
12. **Factors affecting the practice of testicular Self –Examination**
13. Touching one’s testes is embarrassing? Yes/No
14. Fear of discovering a lump? Yes/No
15. Lack of knowledge on how to do Testicular Self–Examination? Yes/No
16. Touching one’s testes is a sin? Yes/No
17. Doing Testicular Self–Examination is time-consuming? Yes/No
18. Testicular Self–Examination should be done by older men? Yes/No
